# Mendelian Randomization Study With Clinical Follow‐Up Links Metabolites to Risk and Severity of Pulmonary Arterial Hypertension

**DOI:** 10.1161/JAHA.123.032256

**Published:** 2024-03-08

**Authors:** Elham Alhathli, Thomas Julian, Zain Ul Abideen Girach, A. A. Roger Thompson, Christopher Rhodes, Stefan Gräf, Niamh Errington, Martin R. Wilkins, Allan Lawrie, Dennis Wang, Johnathan Cooper‐Knock

**Affiliations:** ^1^ Sheffield Institute for Translational Neuroscience (SITraN), University of Sheffield Sheffield UK; ^2^ Division of Evolution, Infection and Genomics, School of Biological Sciences The University of Manchester Manchester UK; ^3^ Department of Nursing, Faculty of Applied Medical Sciences Taif University Taif Saudi Arabia; ^4^ Department of Infection, Immunity and Cardiovascular Disease University of Sheffield Sheffield UK; ^5^ Department of Computer Science University of Sheffield Sheffield UK; ^6^ National Heart and Lung Institute, Imperial College London London UK; ^7^ Department of Respiratory Medicine University of Cambridge Cambridge UK; ^8^ Singapore Institute for Clinical Sciences, Agency for Science, Technology and Research (A*STAR) Singapore Republic of Singapore

**Keywords:** homostachydrine, Mendelian randomization, metabolome, pulmonary arterial hypertension, serine, Epidemiology, Pulmonary Hypertension, Genetics, Metabolism

## Abstract

**Background:**

Pulmonary arterial hypertension (PAH) exhibits phenotypic heterogeneity and variable response to therapy. The metabolome has been implicated in the pathogenesis of PAH, but previous works have lacked power to implicate specific metabolites. Mendelian randomization (MR) is a method for causal inference between exposures and outcomes.

**Methods and Results:**

Using genome‐wide association study summary statistics, we implemented MR analysis to test for potential causal relationships between serum concentration of 575 metabolites and PAH. Five metabolites were causally associated with the risk of PAH after multiple testing correction. Next, we measured serum concentration of candidate metabolites in an independent clinical cohort of 449 patients with PAH to check whether metabolite concentrations are correlated with markers of disease severity. Of the 5 candidates nominated by our MR work, serine was negatively associated and homostachydrine was positively associated with clinical severity of PAH via direct measurement in this independent clinical cohort. Finally we used conditional and orthogonal approaches to explore the biology underlying our lead metabolites. Rare variant burden testing was carried out using whole exome sequencing data from 578 PAH cases and 361 675 controls. Multivariable MR is an extension of MR that uses a single set of instrumental single‐nucleotide polymorphisms to measure multiple exposures; multivariable MR is used to determine interdependence between the effects of different exposures on a single outcome. Rare variant analysis demonstrated that loss‐of‐function mutations within activating transcription factor 4, a transcription factor responsible for upregulation of serine synthesis under conditions of serine starvation, are associated with higher risk for PAH. Homostachydrine is a xenobiotic metabolite that is structurally related to l‐proline betaine, which has previously been linked to modulation of inflammation and tissue remodeling in PAH. Our multivariable MR analysis suggests that the effect of l‐proline betaine is actually mediated indirectly via homostachydrine.

**Conclusions:**

Our data present a method for study of the metabolome in the context of PAH, and suggests several candidates for further evaluation and translational research.

**Nonstandard Abbreviations and Acronyms**
ATF4: activating transcription factor 4
BH: Benjamini–Hochberg
FDR: false discovery rate
IVW: inverse‐variance weighted
LOF: loss of function
MR: Mendelian randomization
MVMR: multivariable Mendelian randomization
PAH: pulmonary arterial hypertension
REVEAL: Registry to Evaluate Early and Long‐Term PAH Disease Management

**Research Perspective**

**What Is New?**

- Mendelian randomization screen of the metabolome from genome‐wide association studies totaling >100 000 participants concludes that serum serine is protective against pulmonary arterial hypertension (PAH), and serum homostachydrine increases risk of PAH.
- To validate these findings, we directly measured plasma serine and homostachydrine in an independent cohort of 449 patients with PAH; strikingly, both metabolites were significantly associated with measures of clinical severity with a consistent direction of effect, and homostachydrine had a significant effect on PAH survival.

**What Question Should Be Addressed Next?**

- Follow‐up analysis suggest that both serine and homostachydrine may be acting via modulation of energy metabolism, but this will require further evaluation.
- Our findings could lead to guidance cautioning against the consumption of certain food products containing homostachydrine, or increasing serine consumption, for patients with PAH.

Pulmonary arterial hypertension (PAH), formerly known as primary pulmonary hypertension, is a rare life‐threatening disorder of the pulmonary circulation. It is an archetypal complex disease caused by the interaction of risk genotypes with the environment.^1^ Significant advances have been made to understand genetic and environmental risk factors for PAH,^2^ but much is still unknown, which limits efforts to develop effective treatments. Certain genetic susceptibility genes have been identified, but it is currently not possible to predict who will be affected or when the disease will occur.

The metabolome is an integrator of genetic and environmental factors; it reflects the state of tissues and cells and can alter their function. As such, the metabolome is a good candidate to explore the biological mechanisms underpinning PAH, and indeed there is evidence for the role of the metabolome in the risk and severity of PAH. For example, Rhodes et al^3^ examined 686 biological metabolites in 365 individuals with idiopathic/heritable PAH, 121 healthy controls, and 139 disease controls using ultraperformance liquid chromatography–mass spectrometry. Sets of 20 to 50 metabolites distinguished patients with PAH from controls and disease controls, and correlated with disease severity as measured by survival. Expanding this investigation to patients with chronic thromboembolic pulmonary hypertension demonstrated comparable metabolic abnormalities despite their different underlying biological dysfunction; interestingly, these patients also experienced considerable restoration of their metabolic profiles after therapeutic pulmonary thromboendarterectomy surgery.^4^

Mendelian randomization (MR) is an example of a method for causal inference whereby a significant relationship implies a causal effect of the exposure of interest on the disease trait, providing a series of assumptions are met.^5^, ^6^ First, the instrumental single‐nucleotide polymorphisms (SNPs) must be robustly associated with the exposure. Second, the SNPs must be associated with the disease risk outcome only through the exposure of interest, rather than affecting the outcome via multiple pathways or exposures. Third, the SNPs must not be associated with confounders. In practice, it is often difficult to be sure that all assumptions are met, and in particular it is difficult to exclude the influence of confounders that are genetically correlated with both the exposure and the outcome. As a result, follow‐up orthogonal analyses, particularly those including direct measurement of nominated exposures, are a useful means of supporting an MR result.

Genetic variation is fixed at conception, and therefore SNPs, which form the instruments used in MR, can be considered upstream of adult‐onset disease^7^ such as PAH. We propose that previous studies of the metabolome in PAH have suffered from limited power because of relatively small sample size for the effect sizes they are trying to measure; as an example, the study described above highlighted a panel of metabolites^3^ but was underpowered to implicate individual metabolites in PAH pathogenesis. This is largely a result of a requirement for direct measurement of metabolite concentrations in serum from a large number of patients with PAH. By using MR we overcome this key bottleneck by using genetic correlates to infer metabolite concentrations within PAH patients, thereby negating the requirement for direct measurement. By using different populations to evaluate exposure and outcome, so‐called 2‐sample MR, can achieve larger sample sizes without the need to perform all measurements in the same sample set.^5^ In the same way, MR also alleviates any bias in the selection of subjects, which often hampers observational studies.^5^ We have previously used MR to perform a search for metabolites linked to disease, and here we aimed to apply the same methodology to PAH. We have supported our findings through a series of conditional and orthogonal approaches including direct measurement of serum metabolites in a clinical cohort of patients with PAH. The findings of our study are summarized in Figure 1. Our work opens important new avenues in the search for an understanding of the biological mechanisms underpinning PAH.

**Figure 1:**
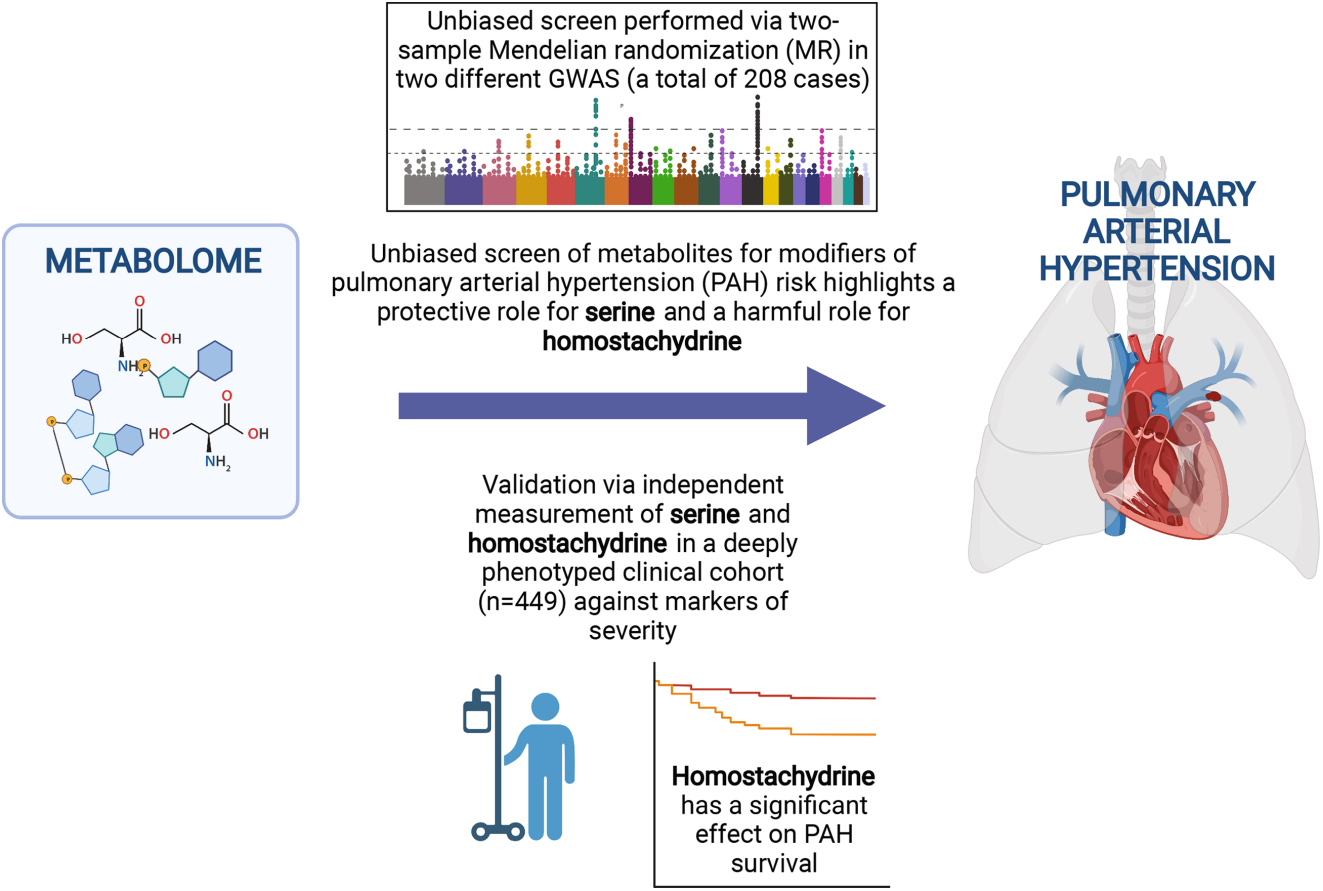
Overview of the metabolome‐wide screen for metabolites that modify risk of pulmonary arterial hypertension (PAH). We performed a 2‐sample Mendelian randomization (MR) screen of 575 metabolites to identify those causally linked to PAH in a FinnGenn cohort. Metabolites that the passed Benjamini–Hochberg false discovery rate multiple testing correction were further evaluated by robust MR measures, sensitivity analysis, and in a validation FinnGenn PAH genome‐wide association study (GWAS). Finally, we directly measured these metabolites in an independent UK PAH cohort of 449 patients and demonstrated a correlation with disease severity.

## Methods

The authors declare that all supporting data are available within the article and its online supplementary files. Data sets used in our analysis are cited below including reference to ethical approval, but no new institutional review board approval or informed consent was required for this article.

### Exposure and Outcome Genome‐Wide Association Study

We used 2‐sample MR to determine whether metabolite exposures are causally linked to the risk of PAH in a FinnGen PAH genome‐wide association study (GWAS) of 125 cases and 162 837 controls (https://www.finngen.fi/en). A more recent overlapping FinnGen PAH GWAS of 208 cases and 243 756 controls was used to confirm metabolites, which passed Benjamini–Hochberg (BH) false discovery rate (FDR) multiple testing correction in the first GWAS.^8^

We performed an analysis of the effect of 575 serum metabolites on PAH risk using publicly available GWAS studies of the serum metabolome.^9^, ^10^ The 2 source studies differed in sample size and methodology: Kettunen et al^10^ employed nuclear magnetic resonance spectroscopy to quantify metabolites in serum samples from up to 24 925 individuals, whereas Shin et al^9^ employed liquid‐phase and gas chromatography coupled with tandem mass spectroscopy in serum from 7824 individuals. The sample population for both studies was almost exclusively European; both studies included ≈2000 individuals from the Cooperative Health Research in the Region of Augsburg cohort. The different methodologies employed were complementary: Nuclear magnetic resonance is more easily applied in a high‐throughput manner, but sensitivity is lower than mass spectroscopy‐based quantification.^11^ A total of 16 metabolites (4% of the total number) were measured in both source studies not including any of our 5 top candidates.

As a further confirmation, we used a new GWAS study of the metabolome^12^ in 50 000 Europeans from the INTERVAL study (https://www.intervalstudy.org.uk).^13^ Plasma concentrations of serine were measured using the Metabolon HD4 Discovery platform (Metabolon, Durham, NC), which is a liquid chromatography coupled with mass spectroscopy method similar to that used in the Shin et al study.^9^

We performed a second MR screen of the effect of 150 immune traits on PAH risk. Here, we used a separate publicly available GWAS of blood concentration of a set of human immune system traits in 669 female twins from the United Kingdom.^14^ Immune cells were quantified via specific antibodies and flow cytometry.

### Two‐Sample MR

Genetic instruments were selected on the basis of a *P* value threshold of either *P*=5e‐06, *P*=5e‐07, or *P*=5e‐08; the lowest threshold was chosen to ensure >10 instrumental SNPs. When the cutoff is too low, informative instruments will be lost, but when it is too high, noninformative instruments will be introduced and instrument pleiotropy is more likely to occur.^15^ For each metabolite and immune trait studied in our MR screens, we used multiplicative random‐effects inverse‐variance weighted (IVW) estimate for significance testing, as this method has the greatest statistical power,^16^ and it allows us to make accurate causal inferences for >4 instrumental SNPs under the assumption of limited balanced pleiotropy. IVW estimates were FDR^17^ corrected for multiple testing by the BH method^17^ assuming that the condition of “positive regression dependency on each one from a subset”^18^ is met; a threshold of FDR<0.05 was applied to identify metabolites that are causally associated with PAH. In practice, it is difficult to be sure that the condition of positive regression dependency on each one from a subset is met, and therefore we also applied both a Bonferroni and a Benjamini–Yekutieli correction for multiple testing, which assume either no interdependence or are agnostic to the nature of dependence between statistical tests.^18^ Independent SNPs were clumped using a stringent cutoff of *R*<0.001 within a 10 000‐kb window in the European reference panel. For clumped SNPs in linkage disequilibrium, the SNP with the lowest *P* value was retained. When an exposure SNP was unavailable in the outcome data set, a proxy with a high degree of linkage disequilibrium (*R* ^2^>0.9) within a European reference population was identified. SNP effects on outcomes and exposures were harmonized so that the beta values are based on the same alleles. To reduce the risk of errors due to strand issues, palindromic alleles with minor allele frequency >0.42 were excluded from the analysis.^15^

### Robust Tests and Sensitivity Analysis

To increase confidence in the IVW results from our unbiased screening, we performed a series of robust MR measures and sensitivity analyses. We used an *F*‐statistic to measure the strength of the association between instrumental SNPs and the exposure of interest. An *F*‐statistic >10 indicates that an SNP‐derived estimate has a bias of <10% of its intragroup variability and signifies an acceptable instrument. Pleiotropy occurs between SNPs for which the difference in effect size for the exposure is not proportional to the difference in effect size for the outcome, and is usually due to a violation of one of the key assumptions underlying MR: the assumption that instrumental SNPs should be associated with the outcome only through the exposure.^5^ To account for pleiotropy, we removed SNPs for which the *P* value for the association with the outcome was lower than for the association with the exposure of interest. The MR–Egger intercept test was also used to identify directional horizontal pleiotropy. To avoid false positives, we limited our analysis to exposures with >10 instrumental SNPs and used a quantile–quantile plot to determine whether the median *P* value was significantly inflated. A test with too few SNPs gives excessive weight to each individual SNP.^19^ As IVW estimates are vulnerable to pleiotropic SNPs, we used Cochran's *Q* test (*P*>0.05) as a sensitivity measure to detect heterogeneity indicating pleiotropy. Moreover, radial MR^20^ was used to remove statistically significant outlier SNPs. The *I* ^2^ statistic was used to measure the heterogeneity between variant‐specific causal estimates, with a low *I* ^2^ indicating bias toward the null hypothesis.^21^ A leave‐one‐out analysis was applied to identify results in which ≥1 SNPs exert a disproportionate effect. TwoSampleMR (version 0.5.6), Mendelian Randomization (version 0.5.1) and RadialMR (version 1.0) R packages were used for all MR analyses (R Foundation for Statistical Computing, Vienna, Austria).

### Clinical Validation

We evaluated the serum metabolites that were nominated by our 2‐sample MR study using measurements of the same molecules within plasma samples from the UK PAH cohort^3^, ^4^ including 449 PAH patients. In this cohort, metabolic profiling was performed using ultra‐performance liquid chromatography–mass spectrometry.^3^, ^4^ We examined the concentration of serine and homostachydrine and tested for correlation between identified candidates and clinical measures of PAH severity including survival. In all analyses, we adjusted for other covariates such as age, sex, body mass index (BMI), population ancestry (Asian, Black, White, not stated, and other) and geographic location of the sample collection.

Clinical measures of severity in the UK PAH cohort included World Health Organization functional class; NT‐ proBNP (N‐terminal pro‐B‐type natriuretic peptide), which correlates with myocardial stress and provides prognostic information; 6‐minute walking distance; and a series of measurements taken at right heart catheterization including pulmonary arterial wedge pressure, pulmonary vascular resistance, right atrial pressure, cardiac index, cardiac output, and venous oxygen saturation. We also included the percentage of predicted forced vital capacity as a measure of lung function. The Registry to Evaluate Early and Long‐Term PAH Disease Management (REVEAL) is an aggregate score used for prognostication in PAH.^22^ Many of these measures are not specific to PAH but have been associated with clinical outcomes including survival. In the analysis of clinical measurements we used linear/logistic regression adjusted for covariates including age, sex, BMI, ethnicity, and geographic location of the sample collection. In the analysis of survival, we applied Cox regression adjusted for age, sex, BMI, ethnicity, and geographic location of the sample collection. Kaplan–Meier curves were plotted by dividing patients with PAH on the basis of their serum metabolite concentrations into equal‐sized percentiles.

### Multivariable MR

Multivariable MR (MVMR) is a conditional MR analysis technique^23^ that we used to determine whether the protective effect of serine was mediated via regulation of immune cell numbers and whether the toxic effect of homostachydrine was detectable via coffee consumption or mediated via l‐proline betaine. The *P* value cutoffs used to select instrumental SNPs for each exposure were chosen so as to achieve adequate instrument strength for both exposures (conditional *F*‐statistic >10 for each exposure). Reported results showed no evidence of instrument heterogeneity (Cochran's *Q* test *P*>0.05). Exposures were derived from independent cohorts and therefore a correction for the covariance between the effect of the genetic variants on each exposure was not necessary. MVMR was implemented using the MVMR (version 0.3) and MendelianRandomization (version 0.5.1) R packages.

### Rare Variant Analysis

MR is based on common genetic variants (minor allele frequency >1%), which are largely orthogonal to large‐effect rare missense variants. We therefore used the AstraZeneca PheWAS portal^24^ to analyze the Union Primary Pulmonary Hypertension data set (361 675 controls and 578 PAH cases) to determine whether rare variants within enzymes responsible for metabolism of our candidate metabolites are also associated with risk of PAH. Identified rare variants with a common biological effect were collapsed into a single Fisher's exact 2‐sided test to determine whether the burden of variants is different in PAH cases and controls. We focused on loss‐of‐function (LOF) variants including protein‐truncating variants, missense variants, and in‐frame insertions and deletions that are damaging to the protein (Rare Exome Variant Ensemble Learner score ≥0.25)^25^ that were rare (minor allele frequency <0.001 if protein‐truncating or minor allele frequency <0.0005 if non–protein‐truncating, in both UK Biobank and GnomAD^26^); in addition, we considered that variants were high‐quality variant calls on the basis of coverage, mapping quality, genotype quality, and Hardy–Weinberg equilibrium (ptvraredmg as described in Wang et al^24^).

## Results

### Metabolome‐Wide MR Causally Associates 5 Metabolites With PAH Risk

We hypothesized that serum metabolites may act upon pulmonary tissue to modify the biological processes leading to PAH. We used MR to perform a study of the entire set of serum metabolites to determine which metabolites are causally related to PAH. To achieve this, we obtained genetic variants associated with the serum concentration of 575 metabolites in 7284 individuals.^9^, ^10^ Next, we applied a series of MR tests in which the exposure was formed from metabolite‐associated genetic variants, and genetic liability to PAH was the outcome measure. Genetic associations with PAH were determined within the FinnGen cohort consisting of 125 patients with PAH and 162 837 controls.^8^

**Figure 2:**
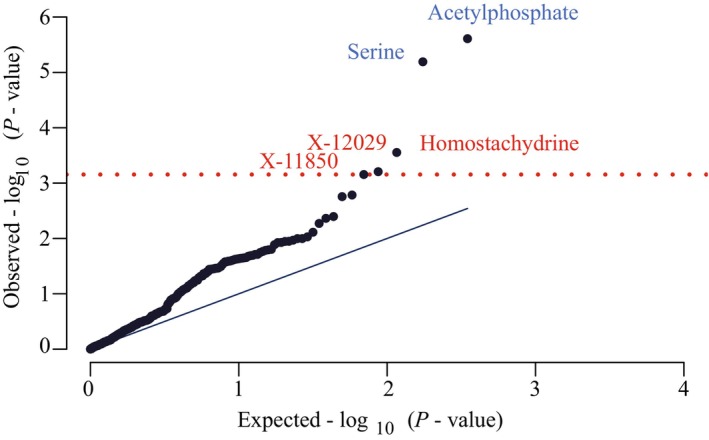
Five metabolites were significantly associated with risk of pulmonary arterial hypertension (PAH) by Mendelian randomization. Quantile–quantile plot of inverse‐variance weighted *P* values demonstrates that 5 metabolites passed a Benjamini–Hochberg false discovery rate multiple testing correction (red line). Blue text represents a protective association, and red text represents a harmful association.

**Figure 3:**
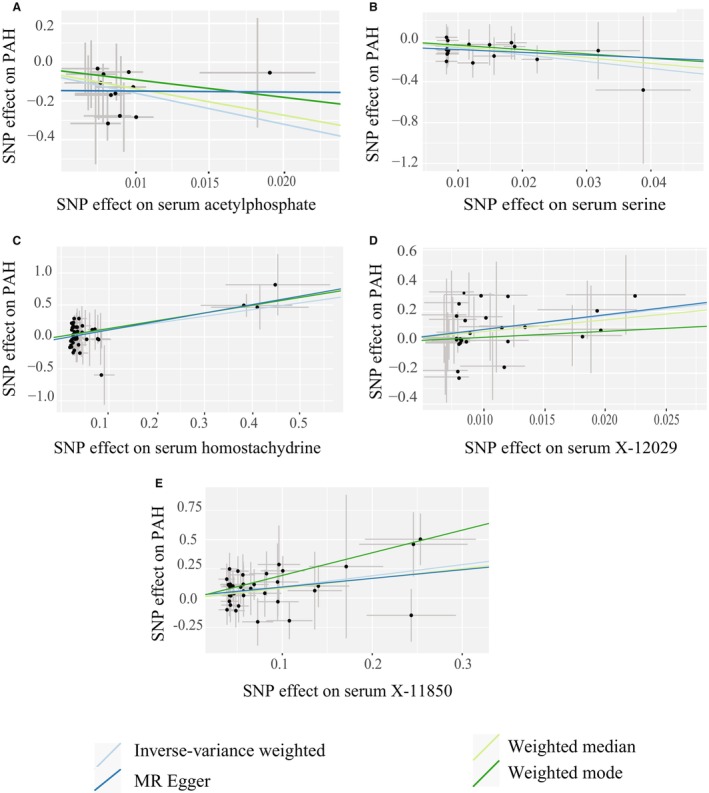
Two‐sample Mendelian randomization (MR) tests for a causal effect of metabolites on risk of pulmonary arterial hypertension (PAH). Scatter plots demonstrate a significant association of serum metabolite concentrations with risk of PAH including in robust MR tests for acetylphosphate (**A**), serine (**B**), homostachydrine (**C**), X‐12029 (**D**), and X‐11850 (**E**). Each point represents the effect size (β) and standard errors for each single‐nucleotide polymorphism (SNP)–outcome relationship.

The complete results of our full metabolome screen are shown in Table S1. After BH FDR correction of the IVW estimate *P* value, 5 metabolites were significantly associated with risk of PAH. Two metabolites were protective against PAH: acetylphosphate (IVW *P*=2.46e‐06, β=−16.06, SE=3.4) and serine (IVW *P*=6.43e‐06, β=−6.7, SE=1.49; Figures 2 and 3A and 3B). Three metabolites increased the risk of PAH: homostachydrine (IVW *P*=0.0003, β=1.09, SE=0.3), X‐12029 (IVW *P*=0.0006, β=8.9, SE=2.6), and X‐11850 (IVW *P*=0.0007, β=0.95, SE=0.28) (Figures 2 and 3C through 3E). Based on the sensitivity tests performed, none of these tests was invalidated by instrument pleiotropy or weak instruments (Table 1). Acetylphosphate was also significantly associated with PAH risk by the weighted median estimate (*P*=0.04, β=−13.7), and X‐11850 was significant by the weighted mode estimate (*P*=0.02, β=1.93). Only homostachydrine was significant in multiple robust MR estimates (weighted median *P*=0.03, β=1.28; MR Egger *P*=0.003, β=1.38; weighted mode *P*=0.006, β=1.26, Table 1), which lends significant support to the validity of this metabolite as a mediator of toxicity leading to PAH.

**Table 1:** Robust MR Measures for the Effect of Significant Metabolites on Risk of PAH

Test	Serine	Acetylphosphate	Homostachydrine	X‐12029	X‐11850
IVW *P* value	6.43e‐6*	2.46e‐06*	0.0003*	0.0006*	0.0007*
IVW beta	−6.7*	−16.06*	1.09*	8.9*	0.95*
Weighted median *P* value	0.13	0.04*	0.03*	0.07	0.1
Weighted median β	−5.5	−13.7*	1.28*	7.6	0.84
Egger *P* value	0.69	0.98	0.003*	0.33	0.23
Egger β	−2.68	−0.51	1.38*	9.58	0.74
Weighted mode *P* value	0.36	0.4	0.006*	0.5	0.02*
Weighted mode β	−4.2	−9.08	1.26*	3.9	1.93*
Mean *F* test	35	25	18	22	18
IVW Cochran's *Q* test *P* value	1	0.93	0.8	0.8	0.9
Radial MR outlier SNPs	0	0	2	1	4
Egger intercept test	0.51	0.53	0.3	0.9	0.7
*I* ^2^	0.97	0.96	0.94	0.96	0.95
Number of SNPs LOO>0.05	0	0	0	0	0
Total number of SNPs	17	11	42	25	36
Number of pleiotropic SNPs	0	0	0	0	0

IVW indicates inverse‐variance weighted; MR, Mendelian randomization; PAH, pulmonary arterial hypertension; and SNP, single‐nucleotide polymorphism.

* Statistically significant results.

There is some uncertainty as to which multiple testing correction to apply to the IVW estimate *P* value given the correlated nature of serum metabolite concentrations. The BH correction assumes the condition of “positive regression dependency on each one from a subset,”^18^ but it is difficult to be certain that this condition is fulfilled. The Benjamini–Yekutieli correction is agnostic to the nature of dependence between tests,^18^ and a Bonferroni correction assumes no interdependence. Under either the Benjamini–Yekutieli or Bonferroni multiple testing correction, then, only serum concentrations of acetylphosphate and serine are significantly associated with risk of PAH at a threshold corrected *P*<0.05.

### Validation in Additional GWAS Supports the Validity of Identified Metabolites

To validate the set of metabolites we associated with risk of PAH, we obtained a different larger FinnGen GWAS consisting of 208 PAH cases and 243 756 controls^8^ that overlaps with our original sample set. Repeating the MR tests for the association of serum metabolite concentration with risk of PAH in this data set confirmed our previous results for serine, acetylphosphate, and homostachydrine but not for X‐12029 and X‐11850. In this analysis, both acetylphosphate (IVW *P*=0.0026, β=−11.3, SE=3.76; Figure S1A) and serine (IVW *P*=0.0004, β=−5.23, SE=1.49; Figure S1B) were significantly protective against PAH, while homostachydrine increased risk of PAH (IVW *P*=0.004, β=0.67, SE=0.24; Figure S1C). X‐12029 (IVW *P*=0.1, β=3.6, SE=2.2) and X‐11850 (IVW *P*=0.056, β=0.5 SE=0.26) were not significantly associated with the increased risk of PAH but maintained the same direction of effect. Serum serine was also significantly protective against PAH when measured in a different GWAS of plasma serine concentration^12^ using the original FinnGen outcome GWAS and using the same instrumental SNPs (IVW *P*=0.001, β=−1.08, SE=0.339; Figure S1D). After these analyses X‐12029 and X‐11850 were excluded from further analysis, as these results were deemed to be nonreproducible.

### Direct Measurement of Serine and Homostachydrine Supports a Link to PAH Clinical Severity

MR is a powerful tool that leverages large sample sets. However, MR relies on genetic correlates of measured exposures and outcomes, which is not equivalent to a direct measurement. We therefore sought to further validate our results by direct measurement of metabolites in a set of clinically profiled PAH patients from the PAH UK cohort of 449 patients with PAH including 260 patients with idiopathic PAH.^3^, ^4^ Unlike our MR analysis, this experiment cannot exclude reverse causality; however, where direct measurement supports our MR analyses, we conclude that there is convergent evidence that a particular metabolite modifies biological processes leading to PAH.

**Figure 4:**
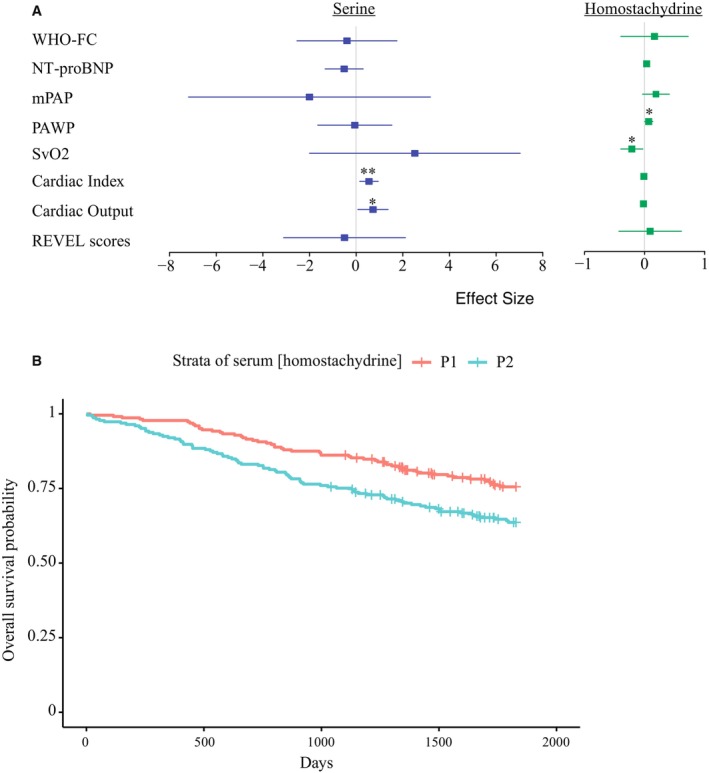
Serum metabolite levels are correlated with clinical severity in the UK pulmonary arterial hypertension (PAH) cohort. **A**, Forest plot to demonstrate linear and logistic regression of biomarkers of PAH severity against plasma concentration of metabolites. Plots indicate mean and 95% CIs. Effect size refers to regression coefficient. All tests were adjusted for age, sex, body mass index, geographic location and population ancestry. **P<0.01, *P<0.05. **B**, Higher serum homostachydrine is associated with a significant reduction in survival (censored at 5 years) in the UK PAH cohort (Cox regression, cor=6.76e‐02, *P*=0.03) after adjusting for age, sex, body mass index, geographic site, and ethnicity. A Kaplan–Meier curve is shown where patients with PAH are divided into subgroups based on the following percentiles of their serum concentration of homostachydrine: P1: <50%, P2: >50%. mPAP indicates mean pulmonary arterial pressure; NT‐proBNP, N‐terminal pro‐B‐type natriuretic peptide; PAWP, pulmonary arterial wedge pressure; REVEL, Rare Exome Variant Ensemble Learner; SvO_2_, venous oxygen saturation; and WHO‐FC, World Health Organization functional class.

In this clinical data set, the plasma concentration of both serine and homostachydrine were significantly correlated with measures of PAH severity after adjusting for age, sex, BMI, population ancestry, and geographic location. Serum serine was positively correlated with measures of cardiac function (Table 2; Figure 4A): cardiac index (linear regression, coefficient=0.56, *P*=0.005) and cardiac output (coefficient=0.73, *P*=0.027) and the correlation with cardiac index remained significant after BH FDR correction. Serum homostachydrine was positively associated with NT‐proBNP (coefficient=0.035, *P*=0.05) and pulmonary arterial wedge pressure (coefficient=0.069, *P*=0.047) and negatively correlated with venous oxygen saturation (coefficient=−0.21, *P*=0.025) (Table 2; Figure 4A). In each case, and even for results that did not reach statistical significance, the direction of effect was consistent with our MR analyses; that is, serine was protective and homostachydrine was harmful. In addition, increased serum homostachydrine was associated with a reduction in survival (Cox regression, coefficient=6.76e‐02, *P*=0.03) after adjusting for age, sex, BMI, population ancestry, and geographic location (Figure 4B). Serum serine concentration was correlated with an increase in survival, but after adjusting for covariates, the results were not significant (Figure S2). Direct measurement of acetylphosphate was not possible, and therefore there was no clinical evidence to support or refute the role of acetylphosphate in PAH; to be cautious, we excluded acetylphosphate from further analysis.

**Table 2:** Linear and Logistic Regression of Biomarkers of PAH Severity Against Plasma Concentration of Metabolites

Variable	Serine		Homostachydrine	
	Coefficient	*P* value	Coefficient	*P* value
WHO‐FC	−3.9	0.72	1.66	0.56
NT‐proBNP	−0.5	0.22	0.035	0.051
6MWD	−0.5	0.22	−2.15	0.07
mPAP	−2	0.45	0.19	0.09
PAWP	−0.05	0.94	0.069	0.047*
SvO_2_	2.5	0.27	−0.21	0.025*
Cardiac index	0.56	0.005*	−0.01	0.19
Cardiac output	0.73	0.027*	−0.017	0.2
PVRdynes	−72	0.48	8.27	0.049*
REVEAL scores	−4.9	0.71	0.09	0.72

All tests were adjusted for age, sex, body mass index, geographic location, and population ancestry. 6MWD indicates 6‐minute walking distance; mPAP, mean pulmonary arterial pressure; NT‐proBNP, N‐terminal pro‐B‐type natriuretic peptide; PAH, pulmonary arterial hypertension; PAWP, pulmonary arterial wedge pressure; PVRdynes, pulmonary vascular resistance in dynes; SvO_2_, venous oxygen saturation; and WHO‐FC, World Health Organization functional class.

* Statistically significant results.

### Homostachydrine Association With PAH Is Independent of Coffee Consumption

Homostachydrine is a xenobiotic metabolite (also known as pipecolic acid betaine) that is highly homologous to l‐proline betaine (also called stachydrine). Initially, it was discovered in leaves of the *Medicago* (alfalfa) and *Achillea* genera. Homostachydrine is also present within frequently used robusta coffee beans.^27^ Indeed, serum homostachydrine has been proposed as a marker of coffee consumption.^28^ We have demonstrated that serum homostachydrine is causally associated with PAH risk and severity; however, it may not be directly related to PAH. Instead, it may act via an intermediate that is also present within coffee. To investigate this possibility, we performed an MR test for the effect of coffee consumption on risk of PAH.

We used a GWAS for questionnaire‐reported coffee consumption consisting of 428 860 individuals.^29^ There was no observed effect of coffee consumption on risk of PAH (IVW *P*=0.87, β=0.27). In addition, we performed a conditional analysis of the effect of homostachydrine on risk of PAH independent of coffee consumption using MVMR. MVMR revealed that the association of homostachydrine with PAH was not invalidated when conditioned on coffee consumption (β=0.97, *P*=0.006). These data suggest that homostachydrine is acting directly and is not serving as a proxy for another component of coffee, although an important caveat is that questionnaire‐reported coffee consumption may be inaccurate.

### Effect of L‐Proline Betaine Effect on PAH Is Dependant on Homostachydrine

Homostachydrine is structurally similar to l‐proline betaine, but it contains a different alkyl chain length and substitution pattern. It is likely that the 2 share a common metabolic pathway and their serum concentrations may be correlated. Interestingly, an increase in serum l‐proline betaine has been associated with a poor prognosis in PAH.^30^ Our MR analysis supports this conclusion: We found a borderline significant toxic effect of l‐proline betaine on PAH (IVW *P*=0.055, β=5.14, SE=2.68). This raised a question as to whether the effects of homostachydrine and l‐proline betaine are independent. To investigate this further, we performed an MVMR in which we discovered that the effect of l‐proline betaine is removed (β=1.8, *P*=0.39) when we conditioned on homostachydrine; whereas homostachydrine was associated with increased risk of PAH (β=2.04, *P*=0.054) even when conditioned on l‐proline betaine. We conclude that the effect of homostachydrine on risk of PAH is independent of the effect of l‐proline betaine, and homostachydrine may even account for previously reported observations regarding the effect of l‐proline betaine on PAH.

### There Is no MR Evidence That the Effect of Serine on PAH Is Mediated via Immunomodulation

We have identified a protective effect of serine on risk and severity of PAH. We interrogated the literature to determine a likely mechanism for this effect. Activated immune cells are rapidly proliferating and are thus dependent on serine to function.^31^ Moreover, patients with PAH exhibit immune system abnormalities.^32^, ^33^, ^34^ We hypothesized that the protective effect of serine on PAH may be mediated via immunomodulation.

To investigate this further, we performed a second MR screen of immune traits^14^ as determinants of PAH risk (Table S2). After BH FDR multiple testing correction, only 2 traits were significantly associated with risk of PAH: the proportion of Vδ1+ T cells (IVW, *P*=0.00006, β=−1.02) and the proportion of CD1c‐ myeloid dendritic cells (IVW, *P*=0.00076, β=0.84). Vδ1+ T cells are tissue‐resident lymphocytes involved in immune surveillance where they receive antigen presentation from dendritic cells including CD1c‐ myeloid dendritic cells. Serum serine was not significantly associated with either of these traits (IVW, *P*>0.05). Nineteen immune traits were nominally (IVW *P*<0.05) associated with PAH risk; but only 2 of these immune traits: the concentration of CD8^+^ T cells (IVW, *P*=0.013, β=0.6), and the concentration of CD3 on double‐negative T cells (IVW, *P*=0.042, β=0.9), were also significantly associated with serum concentration of serine (Table S3). CD8^+^ T cells are cytotoxic, whereas double‐negative T cells are associated with immunomodulation; the notable exception here is CD4^+^ T cells, which suggests that these helper T cells may be relatively less dependent on serine for their function. We applied MVMR to determine whether the effect of serine on PAH was mediated via either of these immune traits; to the contrary, in MVMR the effect of serine on PAH remained significant (*P*<0.05), but both immune traits were nonsignificant when conditioned on serine. Our MR analysis thereby suggests that the effect of serine is independent of immune function and not mediated via CD8 T cells or CD3^+^ double‐negative T cells.

### Rare Variant Analysis Supports the Validity of Serine as Protective Against PAH

If serum serine is protective against PAH, it follows that genetic variants that reduce synthesis of endogenous serine might modify the risk of PAH. Activating transcription factor 4 (ATF4) is a transcription factor that promotes the expression of enzymes involved in endogenous synthesis of serine under conditions of serine starvation^35^ and has previously been linked to a related disease, dilated cardiomyopathy.^36^ We performed rare variant genetic burden testing in a cohort of 578 PAH cases compared with 361 675 controls to determine whether patients with PAH carried an excess of LOF mutations within ATF4 or any of the enzymes under its control (Table 3). There was an excess of LOF mutations within ATF4 in PAH cases compared with controls (Fisher's exact test, odds ratio=3.78, *P*=0.047), which is consistent with our MR data suggesting a protective role for serum serine. There was no evidence for an excess of LOF variants in any of the individual enzymes downstream of ATF4 involved in serine biosynthesis.

**Table 3:** LOF Mutations Within the ATF4 Transcription Factor Involved in Control of Serine Biosynthesis Are Associated With Increased Risk of PAH

Gene	*P* value	Number of cases	Number of controls	OR	Biological role
*ATF4*	0.047*	3 (0.53%)	498 (0.14%)	3.78	TF that upregulates expression of PSPH, PSAT, PHGDH and SHMT2
*PSPH*	>0.1	…	…	…	Serine biosynthesis
*PSAT*	>0.1	…	…	…	
*PHGDH*	>0.1	…	…	…	
*SHMT2*	>0.1	…	…	…	Conversion between glycine and serine

This analysis used whole‐exome sequencing from 578 patients with PAH and 361 675 controls.^24^ Numbers refer to the total number of PAH cases and controls who carried a LOF variant under the ptvraredmg classification.^24^ LOF indicates loss‐of‐function; OR, odds ratio; PAH, pulmonary arterial hypertension; PHGDH, phosphoglycerate dehydrogenase; PSAT, phosphohydroxythreonine aminotransferase; PSPH, phosphoserine phosphatase; SHMT2, serine hydroxymethyltransferase; and TF, transcription factor.

* Statistically significant results.

## Discussion

PAH is an archetypal complex disease determined by gene–environment interactions that is currently incurable. The role of the metabolome in the development of PAH is poorly understood, although groups of metabolites have been observed to associate with PAH risk and severity in previous observational studies.^3^ Here, we have applied a metabolome‐wide screen to search for causal links between serum metabolites and PAH risk, leveraging the significant statistical power provided by 2‐sample MR. As a comparison with the cited study of Rhodes et al,^3^ which used 365 patients with PAH and 121 controls, the increase in same size in our 2‐sample MR screen (125 patients with PAH and 162 837 controls) achieves equal power after multiple testing correction, to detect a 20% smaller change in concentration of an individual metabolite.^37^ We have previously used MR to perform a similar search for metabolites that determine risk for amyotrophic lateral sclerosis,^15^ Alzheimer disease,^38^ and age‐related macular degeneration.^39^

We have linked 3 metabolites to the risk of PAH using MR in 2 different GWASs for PAH: acetylphosphate, serine, and homostachydrine. Serine and homostachydrine are supported by orthogonal analysis via direct measurement of serum metabolites in a clinical cohort of patients with PAH. In our MR results, higher serum serine reduces risk for PAH, and in the clinical cohort, higher serum serine was associated with reduced PAH severity and increased survival. In contrast, in our MR results, higher serum homostachydrine increased risk for PAH, and in the clinical cohort, higher serum homostachydrine was associated with increased PAH severity and reduced survival. The orthogonal nature of the tests we have applied provides significant evidence that we have identified new biological mechanisms underpinning PAH.

Serine biosynthesis has been previously implicated in dilated cardiomyopathy via the transcription factor ATF4^36^; dilated cardiomyopathy and PAH may share underlying pathobiology, particularly related to vascular remodeling.^40^ Our rare variant analysis associated LOF mutations within ATF4 with increased risk of PAH, which supports a role for this pathway in PAH. In the study of the effect of serine biosynthesis on dilated cardiomyopathy,^36^ positive treatment effects were correlated with correction of mitochondrial respiration chain defects, suggesting a boost to energy production. Our analyses suggest that serine is not linked directly to PAH via immunomodulation and we conclude that a role in energy metabolism is more likely. Promoting endogenous serine synthesis via the activity of ATF4 may be a novel therapeutic strategy for PAH.

Homostachydrine is a xenobiotic metabolite that is exclusively produced in plants including within robusta coffee beans. Homostachydrine has been proposed as a marker of quality for coffee, which is thought to be improved by a higher proportion of arabica coffee beans.^27^ Indeed, serum homostachydrine has been proposed as a marker of coffee consumption.^28^ However, our analysis suggests that the effect of homostachydrine on PAH is independent of coffee consumption and therefore not likely to be a by‐product of some other component of coffee.

Homostachydrine is structurally similar to l‐proline betaine, and our MR analysis suggests that l‐proline betaine may be indirectly linked to risk of PAH via homostachydrine. Higher fasting blood concentrations of l‐proline betaine have been linked to poor prognosis in PAH, including a rise in NT‐proBNP, which is an important biomarker of PAH severity.^30^ Unlike homostachydrine, there is some understanding of the specific activities and physiological roles of l‐proline betaine, for example, increased concentrations of l‐proline betaine may disrupt mitochondrial structure and function leading to a toxic energy deficit.^41^ It is possible that this effect on energy metabolism is actually mediated via homostachydrine, and that an energy deficit is responsible for the link between serum homostachydrine and the risk and severity of PAH.

Our study has a number of limitations. First, the study population for the immune GWAS consisted entirely of female twins from the United Kingdom. While the focus on monozygotic and dizygotic twins may improve power for the identification of genetic correlates of immune traits, which will aid our MR work, the differences in the study population from both our PAH and our serine GWAS may have led to false negatives. All studies are based on European individuals, but clearly female twins are a nonrandom selection of the population that are likely to be genetically distinct, and SNP–trait associations may not carry over between GWASs. On this basis, we have confidence in our positive results, but we are less certain of our negative results; that is, we are not certain that there is not an immune basis for the protective effect of serine on PAH risk. Second, while we have addressed a number of possible confounders of the proposed causal relationship between metabolites and PAH risk, it is possible that additional confounders were missed. We have attempted to minimize this possibility through the application of multiple orthogonal analyses and the use of several independent cohorts. Third, the sample size of PAH cases in both the FinnGen GWAS and the UK clinical study is relatively small; moreover, the 2 FinnGen GWASs are not independent. The agreement between the MR and clinical analyses is significant, but it is possible that our study included false negatives, which will be revealed by future studies with larger sample sizes. Fourth, ATF4 has a number of roles independent of serine, including in the integrated stress response.^42^ Further experimental study is necessary to determine whether the effect of ATF4 LOF on PAH can be corrected by exogenous addition of serine. Finally, in the initial MR study of serum metabolites as they relate to risk of PAH, homostachydrine passed the BH multiple testing correction but not the Benjamini–Yekutieli or the Bonferroni corrections, which make fewer assumptions about interdependence between statistical tests. In the use of the BH correction for multiple testing when using the IVW test to study the entire metabolome as it relates to risk of disease, we are following a precedence in the literature,^43^, ^44^, ^45^, ^46^ but as a result we can be less confident that the homostachydrine result is a true positive. However, our follow‐up analyses, including robust MR tests and study of PAH severity in an independent clinical cohort all support the idea that serum homostachydrine is linked to risk and severity of PAH.

In conclusion, we have demonstrated the utility of MR for the discovery of disease‐associated metabolites. Our follow‐up analysis in a clinical cohort adds significant orthogonal evidence for our conclusions by adding a direct measurement of serum metabolites in patients with PAH. We conclude that there is consistent evidence for a protective effect of serum serine on PAH and a harmful effect of homostachydrine. Both of these metabolites may be acting via modulation of energy metabolism. Further study is necessary and could lead to guidance cautioning consumption of certain food products containing homostachydrine or increasing serine consumption for patients with PAH.

## Sources of Funding

This work was supported by a Wellcome Trust Fellowship (216 596/Z/19/Z to Dr Cooper‐Knock). Dr Lawrie is supported by a British Heart Foundation (BHF) Senior Basic Science Fellowship (FS/18/52/33808). Dr Rhodes is supported by BHF Basic Science Research Fellowships (FS/15/59/31839 & FS/SBSRF/21/31025) and Academy of Medical Sciences Springboard Fellowship (SBF004\1095). Dr Julian is supported by the NIHR Academic Clinical Fellowship. Dr Thompson was supported by a BHF Intermediate Clinical Fellowship (FS/18/13/33281). This work was supported by the NIHR BioResource, which supports the UK National Cohort of Idiopathic and Heritable PAH, the British Heart Foundation (BHF SP/12/12/29836), and the UK Medical Research Council (MR/K020919/1). This work was supported in part by the British Heart Foundation Centre for Research Excellence award RE/18/4/34215. The authors acknowledge the UK National Cohort Study of Idiopathic and Heritable PAH. The researchers would like to acknowledge Deanship of Scientific Research, Taif University for funding this work. The views expressed are those of the authors and not necessarily those of the National Health Service, the NIHR, or the Department of Health and Social Care. Dr Wang is supported by the Academy of Medical Sciences Professorship (APR7_1002).

## Disclosures

None.

## Supporting information

Tables S1–S3
Figures S1–S2
