# Supplementary material for: Mendelian Randomization Study With Clinical Follow‐Up Links Metabolites to Risk and Severity of Pulmonary Arterial Hypertension
Source: J Am Heart Assoc. 2024 Mar 8;13(6):e032256. doi: 10.1161/JAHA.123.032256 (PMC11010003; doi:10.1161/JAHA.123.032256)
Supplement: Supplementary file 1 — Tables S1–S3 Figures S1–S2 [file JAH3-13-e032256-s001.zip › jah39044-sup-0001-Supinfo.pdf]

SUPPLEMENTAL MATERIAL

Table Legends (see separate Excel files):

Table S1. Metabolome-wide two-sample Mendelian randomization (MR) screen results for 560 metabolites as exposures for pulmonary arterial hypertension (PAH) risk with all the sensitivity measures and robust tests results.

Table S2. Two-sample Mendelian randomization (MR) results for a screen of 144 immune traits as exposures for pulmonary arterial hypertension (PAH) risk with all the sensitivity measures and robust tests results.

Table S3. Two sample Mendelian randomization (MR) screen with serine as exposure and 19 immune traits as exposures. Immune traits were chosen because they were significantly (IVW p-value <0.05) associated with risk of pulmonary arterial hypertension (PAH).

Figure S1. Two-sample Mendelian randomization (MR) tests for a causal effect of metabolites on risk of pulmonary arterial hypertension (PAH) in validation GWAS.

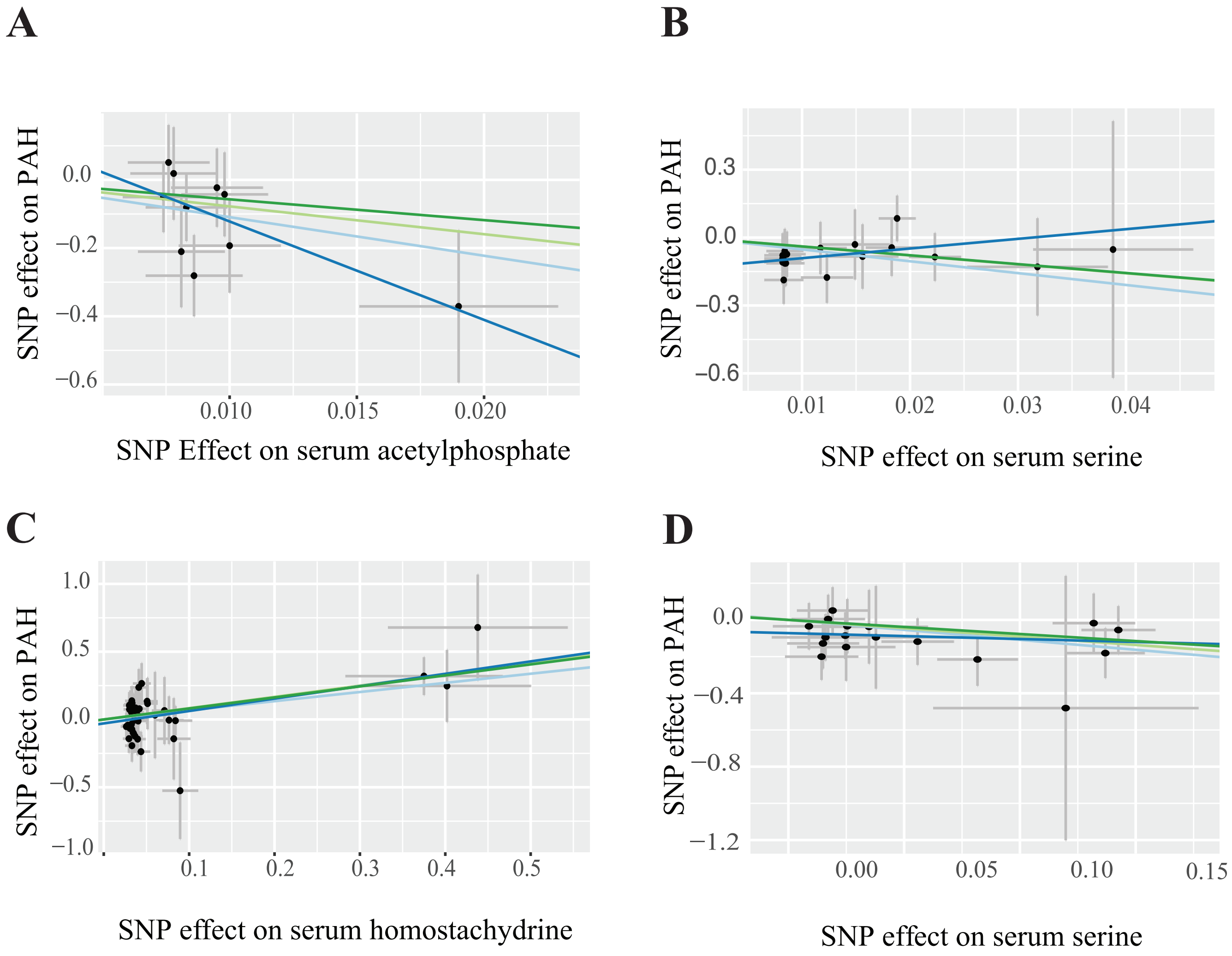

Scatter plots demonstrate a significant association of serum metabolite concentrations with risk of PAH, including in robust MR tests for (A) acetylphosphate (B) serine, (C) homostachydrine, and (D) serine. Compared to the original metabolome screen (A-C) utilise a more recent larger FinnGen PAH GWAS, and (D) utilities a new GWAS for plasma serine12. Each point represents the effect size (beta) and standard errors for each SNP-outcome relationship.

Figure S2. Lower serum concentration of serine is associated with a significant reduction in survival (censored at 5 years) in the UK pulmonary arterial hypertension (PAH) cohort.

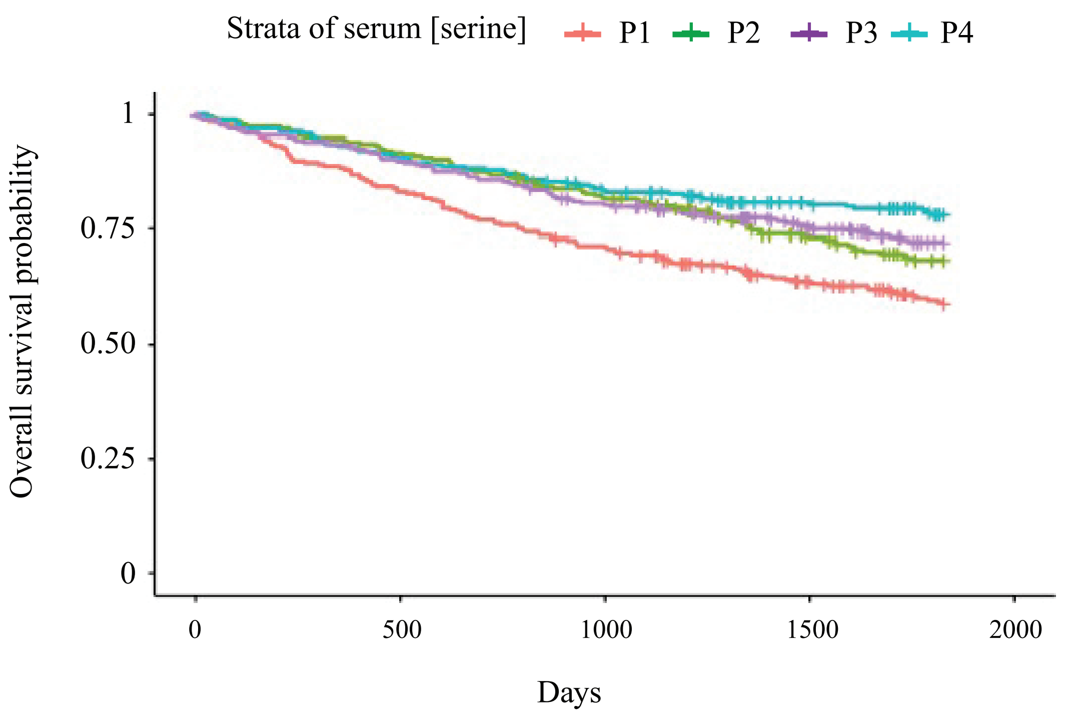

(Cox regression, cor= 6.76e-02, p= 0.03) after adjusting for age, sex, bmi, site and ethnicity. A Kaplan-Meyer curve is shown where PAH patients are divided into subgroups based on the following percentiles of their serum concentration of serine: P1: < 25%, P2: 25%-50%; P3: 50%-75%, P4> 75%.
